# Gradient of Parvalbumin- and Somatostatin-Expressing Interneurons Across Cingulate Cortex Is Differentially Linked to Aggression and Sociability in BALB/cJ Mice

**DOI:** 10.3389/fpsyt.2019.00809

**Published:** 2019-11-15

**Authors:** Sabrina van Heukelum, Floriana Mogavero, Melissa A.E. van de Wal, Femke E. Geers, Arthur S.C. França, Jan K. Buitelaar, Christian F. Beckmann, Jeffrey C. Glennon, Martha N. Havenith

**Affiliations:** Department of Cognitive Neuroscience, Donders Institute for Brain, Cognition and Behaviour, Radboudumc, Nijmegen, Netherlands

**Keywords:** parvalbumin, somatostatin, aggression, social behavior, cingulate cortex, anterior cingulate cortex, midcingulate cortex

## Abstract

Successfully navigating social interactions requires the precise and balanced integration of social and environmental cues. When such flexible information integration fails, maladaptive behavioral patterns arise, including excessive aggression, empathy deficits, and social withdrawal, as seen in disorders such as conduct disorder and autism spectrum disorder. One of the main hubs for the context-dependent regulation of behavior is cingulate cortex, specifically anterior cingulate cortex (ACC) and midcingulate cortex (MCC). While volumetric abnormalities of ACC and MCC have been demonstrated in patients, little is known about the exact structural changes responsible for the dysregulation of behaviors such as aggression and social withdrawal. Here, we demonstrate that the distribution of parvalbumin (PV) and somatostatin (SOM) interneurons across ACC and MCC differentially predicts aggression and social withdrawal in BALB/cJ mice. BALB/cJ mice were phenotyped for their social behavior (three-chamber task) and aggression (resident-intruder task) compared to control (BALB/cByJ) mice. In line with previous studies, BALB/cJ mice behaved more aggressively than controls. The three-chamber task revealed two sub-groups of highly-sociable versus less-sociable BALB/cJ mice. Highly-sociable BALB/cJ mice were as aggressive as the less-sociable group—in fact, they committed more acts of socially acceptable aggression (threats and harmless bites). PV and SOM immunostaining revealed that a lack of specificity in the distribution of SOM and PV interneurons across cingulate cortex coincided with social withdrawal: both control mice and highly-sociable BALB/cJ mice showed a differential distribution of PV and SOM interneurons across the sub-areas of cingulate cortex, while for less-sociable BALB/cJ mice, the distributions were near-flat. In contrast, both highly-sociable and less-sociable BALB/cJ mice had a decreased concentration of PV interneurons in MCC compared to controls, which was therefore linked to aggressive behavior. Together, these results suggest that the dynamic balance of excitatory and inhibitory activity across ACC and MCC shapes both social and aggressive behavior.

## Introduction

Aggression is part of the essential evolutionary survival tool kit for most animals—in specific contexts, e.g., during competition for crucial resources, it can greatly facilitate an animal’s chance of survival (1). However, to avoid negative consequences, aggressive behavior needs to be proportionate to its environmental context. Aggression is therefore subject to strong inhibitory mechanisms, mainly mediated by prefrontal regions (2). If these inhibitory mechanisms fail, aggressive behavior can quickly escalate and lose its adaptive function (3, 4). For instance, aggressive behavior is among the most common causes of referrals to child and adolescent settings (5). In patient populations, aggressive behavior often co-occurs with abnormal social behavior and low empathy, such as in conduct disorder (CD) (6). Similarly, autism spectrum disorder (ASD), though primarily characterized by problems in reciprocal social interaction and communication, often goes along with increased levels of aggression (7, 8). This link between aggression and abnormal social behavior also seems to extend beyond patient populations to preclinical animal models: changes in social behavior in rodents often co-occur with increased aggression (e.g., 9, 10), and the BALB/cJ strain, a popular mouse model of aggressive behavior, also shows reduced levels of social interest (11, 12).

Although human aggression is often seen as a matter of emotional processing, it in fact depends on components of both cognitive and emotional control, ranging from schemas and beliefs driving e.g., threat assessment and behavioral strategies (13, 14) to impulse control and frustration tolerance (15). Given that a nuanced and flexible balance between such processes is needed to generate context-appropriate aggression, it is not surprising that aggression is controlled by a wide network of interconnected cortical and subcortical areas. While ventromedial hypothalamus seems to be the network node most directly tasked with initiating aggressive behavior (16, 17), it is also embedded within a range of context-sensitive control mechanisms. For instance, when ventromedial hypothalamus was stimulated optogenetically, unprovoked attacks increased strongly in the presence of a moving male target, but were markedly reduced in the presence of a stationary or female target (17). Such context-dependent modulation of aggression is thought to depend on prefrontal cortex (6, 18), particularly anterior cingulate cortex (ACC) and midcingulate cortex (MCC). ACC has dense connections to the so-called threat circuit connecting stria terminalis, medial hypothalamus, and dorsal periaqueductal gray matter (19–21), and thus within this circuitry likely regulates threat recognition. MCC, together with the insula, has a pivotal role in orchestrating the salience network consisting of amongst others the dorsolateral prefrontal cortex, the caudate nucleus, the mediodorsal nucleus of the thalamus, and dopaminergic brainstem nuclei (22–24). Within this network, MCC is thought to mediate approach/avoidance decisions during aggressive confrontations by allowing rapid interaction between negative emotions (signaled by ACC and insula) and motor signals (25, 26). This tallies with the general notion that in humans ACC is mainly involved in the regulation of emotion (27, 28), while MCC seems to play an important role in cognition and decision making (e.g., 29, 30). Similar complementary roles of ACC and MCC were recently highlighted in a study by van Heukelum et al. (31), which demonstrated that in BALB/cJ mice an increased volume of ACC and decreased volume of MCC jointly predicted maladaptive aggression compared to a non-aggressive control strain (BALB/cByJ).

Consistent with the extensive overlap between symptoms of aggression and decreased sociability discussed above, these behaviors also recruit overlapping cortical networks: in humans, both patients with CD and with ASD show alterations in the volume of ACC and MCC (32–35). In the BALB/cJ mouse model, van Heukelum et al. (31) recently demonstrated that differential changes in the volume of ACC and MCC predict aggressive behavior in opposite directions: increases in ACC volume but decreases in MCC volume correlated with increases in aggression. This opens up two questions: first, given the decreased sociability in BALB/cJ mice (11), how do anatomical variations in cingulate cortex relate not only to aggression, but also to social behavior in the same mouse model? Second, how is the previously observed overall shift in cortical volumes across cingulate cortex converted into impaired regulation of aggressive behavior? A recent study by Jager et al. (36) suggests that altered inhibitory balance in cingulate cortex may be a key factor: using proton spectroscopy (1H-MRS) and transcriptomics, they showed that aggressive BALB/cJ mice compared to non-aggressive BALB/cByJ mice show a reduced concentration of GABA and altered GABA catabolism in cingulate cortex, with no change in glutamate tone. This is consistent with studies relating whole-brain disturbances of inhibitory interneuron activity to psychiatric symptoms including anxiety, depression, and decreased social behavior in human patients (37–41) and rodent models (42–46). However, it is not well-studied how the behavioral impact of such general changes in cortical inhibition maps onto the relative balance between different interneuron types, as well as onto specific cortical areas, particularly within cingulate cortex.

Here, we aim to expand on these results by examining the specific distribution of two dominant interneuron types—parvalbumin-expressing (PV) and somatostatin-expressing (SOM) interneurons—across the three sub-areas of ACC (A25, A32, A24) as well as MCC, and relating it to both aggressive behavior and social behavior in the BALB/cJ mouse model. By relating performance in a series of behavioral tasks to the quantification of PV and SOM interneuron density across ACC and MCC, we find that the gradient of PV and SOM interneurons across ACC and MCC differentially predicts aggression and sociability in the BALB/cJ mouse. A lack of specificity in the distribution of SOM and PV interneurons across cingulate cortex was mostly associated with decreased sociability, while a decreased concentration of PV interneurons in MCC was most predictive of aggressive behavior. Together, these results demonstrate that the balance of excitatory and inhibitory activity across ACC and MCC can be differentially related to both asocial and aggressive behavior, highlighting the importance of separately studying the sub-areas of cingulate cortex and their individual contributions to behavior.

## Materials and Methods

### Animals

We tested male BALB/cJ (n = 10) and BALB/cByJ (n = 10) mice (8-week old at the start of testing), obtained from Jackson Laboratory (Bar Harbor, ME, USA) in the three-chamber social interaction test (3CT) to assess social behavior and the resident intruder test (RI) to assess aggression. In addition, for both tests, male C57BL/6J (Charles River Laboratories, Erkrath, Germany) mice were used as interaction mice (n = 2) and intruders (n = 20). All mice were housed in an enriched environment (High Makrolon^®^ cages with Enviro Dri^®^ bedding material and Mouse Igloo^®^) and had free access to dry food and water. They were kept at a reversed 12–12 h day-night cycle with sunrise at 7.00 pm. All behavioral testing took place between 9 and 12 a.m. For the 3CT, all BALB mice were housed in groups of three (per group there were two extra mice for housing purposes such that all tested mice lived in groups of three animals). After this test, they were housed individually for 10 days before the RI test was conducted. Intruder mice were housed in groups of five and the two interaction mice were housed together separately from the intruder mice. All animal procedures were conducted in compliance with EU and national regulations as well as local animal use ethical committees (European Directive 2010/63/EU), and approved by the Ethics Committee on Animal Experimentation of Radboud University (RU-DEC number 2014-149).

### Resident Intruder Test

Aggression testing (10 days after the 3CT) was done in the home cage of the BALB/cJ and BALB/cByJ mice in a dark room with red overhead lighting. Behavior was videotaped using an infrared camera (SuperLoLux, JVC). Animals were tested for five consecutive days, and each day each BALB/cJ and BALB/cByJ mouse was confronted in their home cage with a different C57BL/6J intruder mouse [intruder mice were 2 weeks younger than resident mice and also had lower weight; (12)]. Testing started by placing an intruder animal in the home cage of the resident animal, separated by a glass screen to allow for visual and olfactory stimulation for 5 min. Subsequently, the screen was removed and confrontation was allowed for 5 min.

### Three-Chamber Social Interaction Test

A standard 3CT Arena (Noldus) was used and the testing procedure took place in a dark room with red overhead lighting. Behavior was videotaped using an infrared camera (SuperLoLux, JVC). During the first 10 min of the test, the test mice (BALB/cJ or BALB/cByJ mouse) were individually placed in the arena to habituate to the new environment. In both the left and right chamber an empty acrylic cylinder with bars was placed. After these 10 min, an interaction mouse (C57BL/6 mouse, same age as test mouse) was placed in a cylinder, randomly in either the left or right cylinder. Each interaction mouse was used for 10 interactions and testing was spread across 3 days. The order of testing was counterbalanced. For 10 min, test mice were allowed to investigate the arena with the interaction mouse in it.

### Perfusion and Tissue Preparation

BALB/cJ and BALB/cByJ mice were deeply anesthetized with isoflurane (3–5%) and perfused with saline followed by 150 ml of 4% paraformaldehyde solution (PFA) in 0.1 M phosphate buffer (PBS). Brains were removed, fixed overnight in 4% PFA and then kept in 0.1 M PBS at room temperature. One day before cutting, brains were placed in 0.1 M PBS plus 30% sucrose to ensure cryoprotection. Coronal sections (30 µm) were obtained on a freezing microtome (Microm, Thermo Scientific). All sections containing ACC and MCC were placed in running order in containers filled with 0.1 M PBS.

### Interneuron Staining

The same animals as used in the behavioral tasks were used for the immunohistochemical procedures. For immunohistochemical procedures, PV (Swant, PV 27) and SOM (Peninsula Laboratories, T-4103) antibodies were used. For both the PV and SOM staining, a free-floating standard 3, 3’-diaminobenzidine (DAB) peroxidase protocol was carried out at room temperature. Briefly, the sections were rinsed in 0,1 M PBS for 10 min, followed by a 30-min incubation with 0.3% H_2_O_2_. Then, the sections were rinsed three times for 15 min and a 30-min pre-incubation in PBS-BT was performed. The PV (1:40,000) and SOM (1:10,000) antibodies were used as primary antibodies and sections were incubated overnight at room temparature. After the incubation, sections were washed in PBS, followed by 90 min of incubation with Donkey-Anti-Rabbit IgG Biotin SP conjugated (Jackson ImmunoResearch, 1:1,500). Afterwards sections were washed again three times for 15 min and then incubated for 90 min with Vector ABC-Elite (Vector Labs, A and B 1:800) followed by the same washing steps. Then, a pre-incubation with DAB-Ni solution (Vector Labs, without perhydrol) was carried out for 10 min followed by the incubation with DAB-Ni solution with perhydrol. Sections were mounted on gelatin-coated object glasses and dried overnight at 37°C. The sections were then dehydrated in an alcohol series and mounted with Entellan.

### Data Analysis

#### Resident Intruder

**Figure 1:**
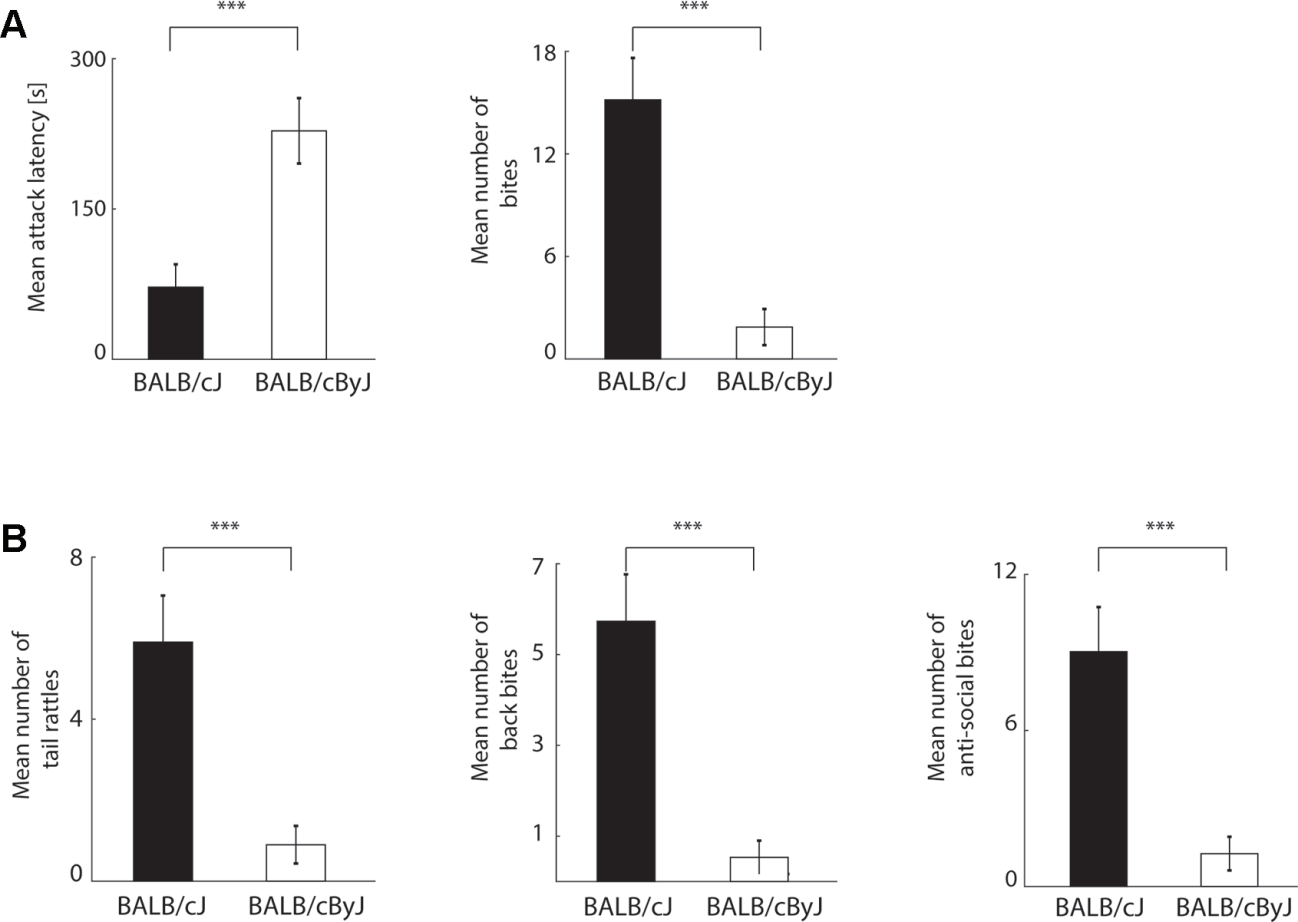
Behavioral read-outs of aggression **(A)** Left panel: average of attack latencies across first three days of resident intruder (RI) testing. Black bar: BALB/cJ mice. White bar: BALB/cByJ mice. Error bars: standard error of the mean (SEM). Right panel: same for number of bites. **(B)** Left and middle panel: average number of tail rattles and back bites (socially acceptable aggression) across first three days of RI testing. Black bar: BALB/cJ mice. White bar: BALB/cByJ mice. Error bars: standard error of the mean (SEM). Right panel: same for anti-social bites (sum of face, neck, and belly bites). ***p < .001.

Attack behavior was scored manually in terms of number of attacks, attack latency, and tail rattles using the program The Observer XT 11 (Noldus). An attack was defined as a bite directed at the back, belly, neck, or face of an intruder (12, 47). All recordings were scored by the same researcher who was blind to the strain of the animal (BALB/cJ and BALB/cByJ mice have the same appearance). A previous study (31) had indicated that spontaneous aggression was best captured by the first three days of the RI test, since even non-aggressive animals learn over time to anticipate intruders entering their home territory, resulting in learned aggression towards an intruder. Since we wanted to examine trait/unprovoked aggression rather than learned/strategic aggression, we therefore included only scores for the first 3 days in the average attack scores shown in **Figure 1**.

#### Three-Chamber Social Interaction Test

The time spent in each of the three chambers and the number of entries into the side chambers, from here on referred to as non-social side (no interaction mouse) and social side (interaction mouse), were analyzed using the program EthoVision XT 9 (Noldus). Time spent sniffing the cylinder in both the non-social and social side were manually scored using the program Observer (Noldus). All recordings were scored by the same researcher who was blind to the strain of the animal and had not scored the corresponding RI data, to avoid any possible bias. In order to classify animals as highly social versus less social based on their performance in the 3CT, we quantified whether they spent significantly more time sniffing the social than the non-social cylinder. This approach was based on the rationale that even animals with no actual social preference could in principle end up randomly spending somewhat more time at the social cylinder than the non-social one. Therefore, in order to verify that an animal clearly preferred the social cylinder, we first established a criterion, calculated as the mean + 2 standard deviations of the time that each group of animals (either BALB/cJ or BALB/cByJ) generally spent sniffing the non-social cylinder. If the time an animal spent sniffing the social cylinder exceeded this criterion, animals were classified as being highly sociable; if not, animals were classified as being less sociable. This does not imply that less sociable animals had absolutely no preference for the social cylinder, just that this preference was too small to be statistically significant.

#### Interneuron Stainings

PV and SOM cells in ACC and MCC were counted using Neurolucida software (MBF Bioscience). The localization of ACC and MCC sections was determined using the newest version of the Paxinos Mouse Brain Atlas (48). We divided the ACC into its subregions A25, A24, and A32 and determined interneuron counts individually for these. The MCC in rodents consists only of A24′ and therefore no subregion division was necessary. To determine the number of PV and SOM cells per mm^2^, contours of the specific regions were drawn for each slice that contained ACC and MCC. Within that area, the number of PV and SOM cells was counted and this number was divided by the surface area, such that all data is expressed as cells per mm^2^. The researcher counting the cells was blind to the group of the animal.

### Statistical Analysis

**Figure 2:**
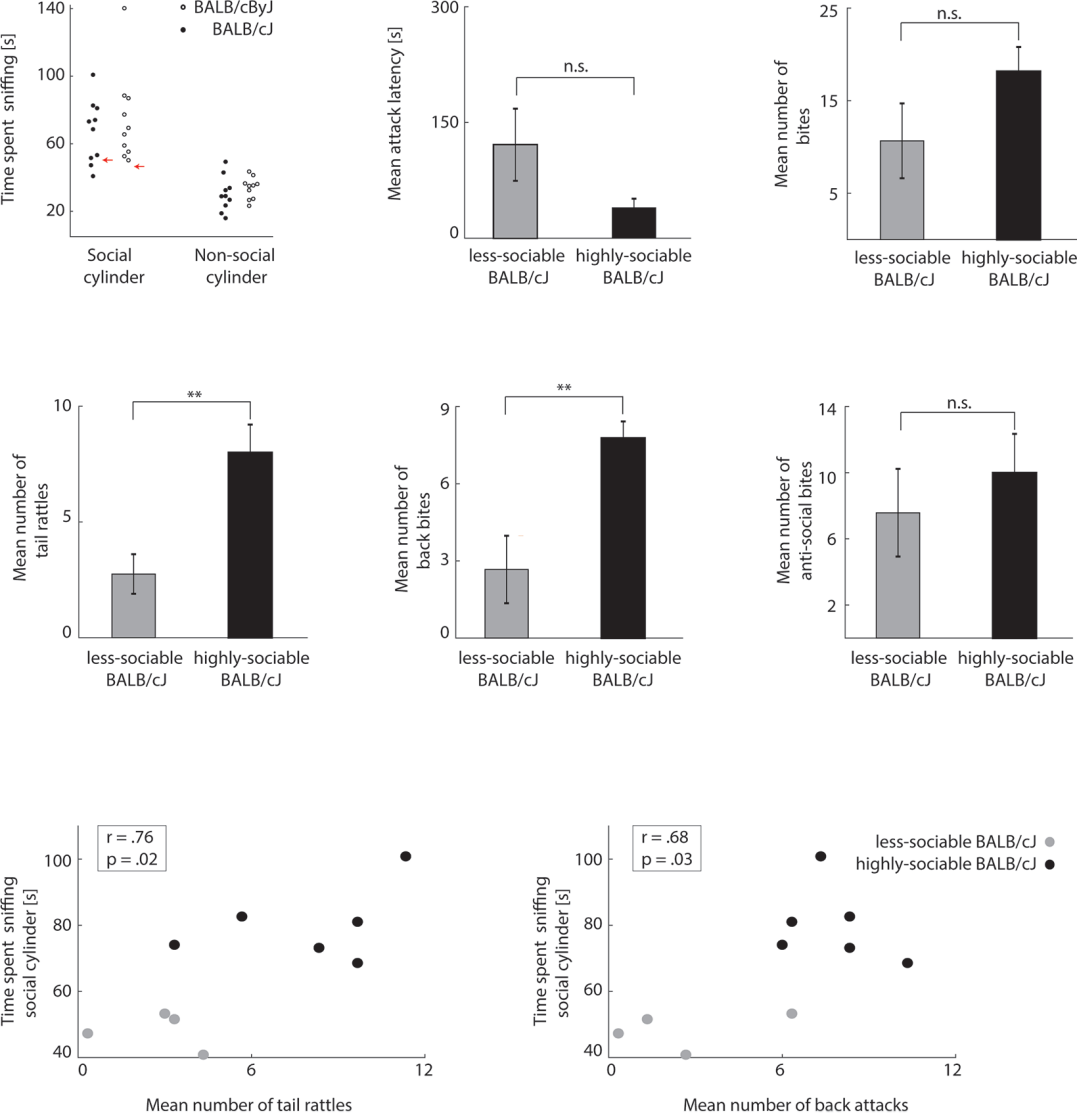
BALB/cJ mice can be split into two groups based on their sociability **(A)** Left panel: sniffing times of social and non-social cylinder in the 3CT test. Black dots: BALB/cJ mice. White dots: BALB/cByJ mice. Red arrows indicate average + 2 standard deviations of non-social cylinder sniffing time. Middle and right panel: average attack latency and number of bites of sociable and less-sociable BALB/cJ mice. Black bar: sociable BALB/cJ mice. Gray bar: less-sociable mice. Error bars: standard error of the mean (SEM). **(B)** Same as **Figure 1B**, but for sociable (black) versus less-sociable BALB/cJ mice (gray). **(C)** Correlation between social cylinder sniffing time and socially acceptable bites (left panel: tail rattles, right panel: back bites). Black dots: sociable BALB/cJ mice. Gray dots: less-sociable BALB/cJ mice. **p < .01; n.s. = not significant.

**Figure 3:**
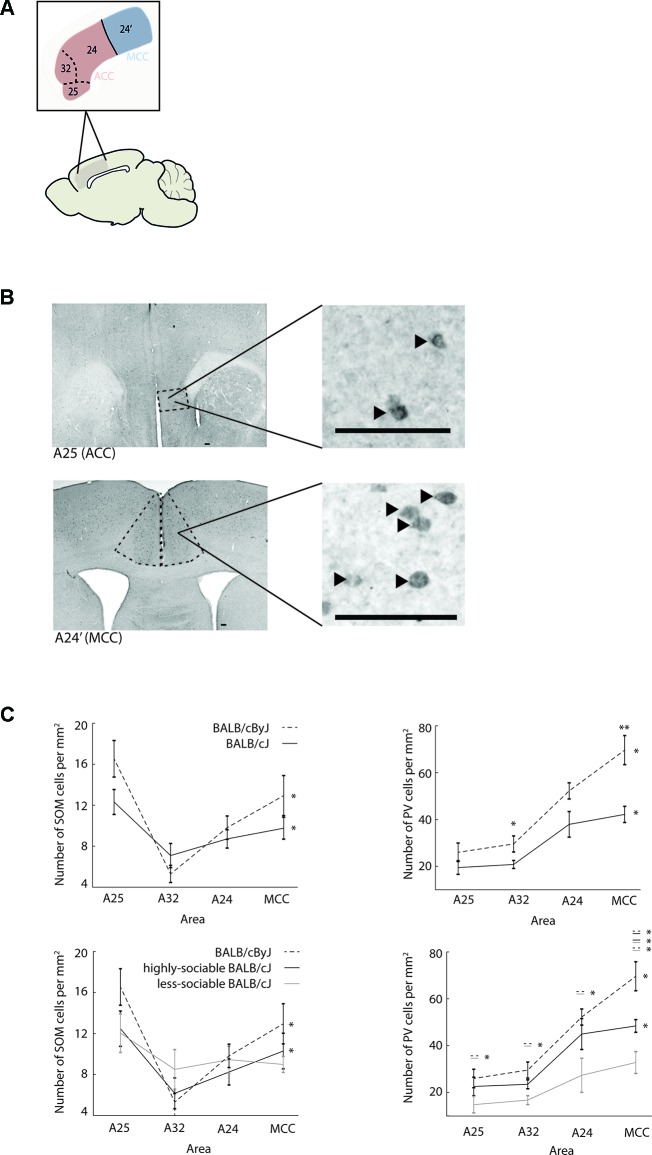
Interneuron populations across anterior cingulate cortex (ACC) and midcingulate cortex (MCC) **(A)** Schematic showing the definition of ACC and MCC used in this study. ACC consists of sub-areas A25, A32, and A24. MCC is not divided into sub-areas in the rodent and consists of area A24′ only. Note: the definition used here is homologous to the definition used in higher mammalian species and does not correspond to the cingulate area 1 *vs*. cingulate area 2 definition. **(B)** First row: macrographs of somatostatin (SOM) interneurons in A25 of ACC (dashed lines), zoom-in shows higher magnification macrograph of SOM interneurons in A25. Scale bar is 100 µm. Black arrow heads point to stained neurons. Second row: same for parvalbumin (PV) neurons in MCC. **(C)** First row: distribution of SOM and PV interneurons across ACC and MCC. Left panel: number of SOM interneurons per mm^2^ in A25, A32, and A24 of ACC and in MCC. Black line: BALB/cJ mice. Black dashed line: BALB/cByJ mice. Error bars: standard error of the mean (SEM). Right panel: same for PV population. Second row: same as first row but plotted separately for sociable (black line) and less-sociable BALB/cJ mice (gray line). *p < .05; **p < .01.

Behavioral data was analyzed with repeated-measure ANOVAs, and t-tests for independent (unpaired) samples were then performed as *post hoc* tests. Strain differences in the number of interneurons were analyzed with non-parametric tests, due to a non-normal distribution of the data, with number of interneurons per mm^2^ as dependent variable and strain as independent variable. The false discovery rate method (49, with a standard q value of .05) was used to correct for multiple comparisons in instances when behavioral differences between strains were tested using more than one behavioral readout (**Figure 1**), when more than two behavioral variables were correlated (**Figure 2**) and when PV/SOM population differences between strains were tested across sub-areas of cingulate cortex (**Figure 3**). We chose the false discovery rate as this method is known to provide a better compromise between type I and type II errors than other correction methods like e.g., the Bonferroni correction (50–52). Correlations shown in the manuscript are Pearson correlations, tested with a two-tailed distribution, and corrected for multiple comparisons with the false discovery rate method. All statistical analyses, including the t-tests and false discovery rate correction, were performed using SPSS23-software (SPSS inc., Chicago, USA).

## Results

### BALB/cJ Mice Are More Aggressive Than BALB/cByJ Mice

We first used the RI test to confirm that BALB/cJ mice engaged in more aggressive behavior than control animals from the BALB/cByJ strain, as indicated by previous studies (12, 31). To investigate differences of aggression across the different days, we performed a repeated measures ANOVA with day as within-subject factor and strain as between-subject factor). In line with our previously published study (31), differences in aggression between the two strains are most pronounced during the first 3 days of testing (see **Supplementary Material Figure 1**). To quantify mean aggression scores per animal, we therefore pooled the data from the first three days of RI testing (**Figure 1**). Indeed, when confronted with an unknown intruder animal, BALB/cJ mice attacked earlier [one-way ANOVA; all presented p-values are corrected with the false discovery rate method; F(1, 18) = 15.43, p = .001, η^2^ = .46] and more often [F(1, 18) = 24.87, p < .001, η^2^ = .58; see **Figure 1A**] than BALB/cByJ mice. Note that aggressive behavior towards an intruder can in principle nevertheless remain within the confines of expected social interaction for (male) mice. For instance, tail rattles and bites to the back of an intruder are typical threat behavior aimed at settling a territorial dispute rather than hurting the opponent in earnest (53–55). In contrast, bites to the face, neck, and belly are physically harmful and potentially lethal, and therefore typically do not take place within the context of a territorial conflict between male mice. To examine whether the elevated aggression in BALB/cJ mice was owed to socially expected and/or anti-social acts of aggression, we separately quantified the frequency of tail rattles and back bites (**Figure 1B**, left and middle panel), and of anti-social attacks (comprised of face, neck, and belly bites; **Figure 1B**, right panel). BALB/cJ mice showed more aggression across both categories: the number of back attacks [F(1, 18) = 22.53, p < .001, η^2^ = .56], tail rattles [F(1, 18) = 16.28, p = .001, η^2^ = .48], as well as anti-social attacks were all increased compared to BALB/cByJ mice [F(1, 18) = 17.95, p < .001, η^2^ = .5]. This demonstrates that consistent with previous results (12, 31), BALB/cJ mice show an elevated level of social *and* anti-social aggression.

### BALB/cJ Mice Form Two Sub-Groups Based on Their Level of Social Interest

To test how aggression and sociability related in the BALB/cJ model, we examined each animal’s sociability by quantifying the time they spent sniffing the social cylinder (containing an unknown mouse) *vs*. the non-social (empty) cylinder in the 3CT (for details, see *Materials and Methods*, all presented p-values are corrected with the false discovery rate). Past research has demonstrated that sniffing time is a more reliable measure for sociability than the time spent in the social *vs*. non-social side (11). As expected, animals generally spent more time sniffing the social than the non-social cylinder [F(1, 18) = 46.96, p < .001, η^2^ = .723]. Interestingly, the amount of social sniffing did not significantly differ between BALB/cJ and BALB/cByJ mice [F(1, 18) = 1.02, p = .326, η^2^ = .054]. However, BALB/cJ mice did appear to show more inter-individual variability in social sniffing time than BALB/cByJ mice. In particular, the lowest four social-sniffing times of the BALB/cJ mice did not significantly exceed the time BALB/cJ mice generally spent sniffing the non-social cylinder (**Figure 2A**, left panel, red arrows indicate mean + 2 stds of non-social cylinder sniffing time). We therefore classified these four animals as less sociable compared to the other six highly sociable BALB/cJ mice, who spent significantly more time sniffing the social cylinder than the non-social one (for details on this analysis, please see the description of the 3CT in the *Data Analysis* section of *Materials and Methods*). Thus, while some BALB/cJ mice (n = 6) appeared to seek a high amount of social contact—in fact a higher amount than most BALB/cByJ mice—others (n = 4) did not seem to substantially prefer the social to the non-social cylinder. Such a division was not apparent for BALB/cByJ mice (**Figure 2A**, left panel, see red arrow on the right). Based on this behavioral divergence, we next explored if BALB/cJ mice with high and low sociability also differed in other neuronal and behavioral traits.

We first examined whether BALB/cJ mice with high and low sociability scores in the 3CT showed differences in aggressive behavior. In terms of overall aggression, this was not the case: highly-sociable and less-sociable BALB/cJ mice did not differ significantly in attack latency and the number of attacks (**Figure 2A**, center and right panel). However, there was a clear behavioral divergence in terms of social versus anti-social forms of aggression: while less-sociable BALB/cJ mice engaged in just as many anti-social attacks as highly-sociable BALB/cJ mice (**Figure 2B**), they displayed reduced levels of socially expected aggression, engaging in fewer tail rattles [F(1, 8) = 16.28, p = .01, η^2^ = .48] and back bites [F(1, 8) = 10.03, p = .013, η^2^ = .56]. Consistent with this, social sniffing time correlated positively with both the number of threats and the number of back bites across all BALB/cJ animals: the more sociable the BALB/cJ mouse, the more social aggression it also displayed (**Figure 2C**). This relationship was absent in BALB/cByJ mice (data not shown, back bites: r = .33, p = .35; tail rattles: r = .24, p = .50; all correlations are Pearson correlations, corrected for multiple comparisons using the false discovery rate method). This implies that in BALB/cJ mice, the general level of sociability generalizes across situations with and without conflict: less-sociable BALB/cJ mice avoided non-aggressive (3CT) *and* aggressive (RI test) social contact with other mice. As a result, we were able to identify a sub-group of BALB/cJ mice that was as, if not more, aggressive as other BALB/cJ mice, but at the same time as, if not more, sociable as control animals from the BALB/cByJ strain. This constellation allowed us to differentially explore potential anatomical underpinnings of aggression and sociability: if an anatomical feature predicted mainly aggressive behavior, it should occur indistinguishably across all BALB/cJ mice; if it predicted mainly sociability, highly-sociable BALB/cJ mice should be indistinguishable from control (BALB/cByJ) mice.

### Somatostatin and Parvalbumin Populations in Anterior Cingulate Cortex and Midcingulate Cortex Relate to Sociability and Aggression

Based on previous literature (6, 56), there is reason to believe that cingulate cortex plays a central role in the regulation of both aggressive and sociable behavior. Specifically, this function is thought to rely on efficient communication between ACC and MCC to generate a balance between external cues and internal motivations (26, 28, 30). We hypothesized that such efficient communication would require precise inhibitory control of intra-cingulate connections. On a structural level, this in turn would require a balanced distribution of inhibitory interneurons across cingulate cortex. To test this hypothesis, we quantified the distribution of two dominant interneuron types—SOM and PV interneurons—across the three sub-areas of ACC (A25, A32, and A24) as well as MCC in the same mice that underwent behavioral testing (**Figure 3**, all presented p-values are corrected with the false discovery rate).

For control animals, both SOM and PV interneurons indeed showed a highly area-specific distribution across cingulate cortex (**Figure 3A**): while SOM interneurons most densely populated A25 and MCC, but were sparse in A32, PV interneuron density increased almost linearly along the anterior-posterior axis of cingulate cortex, peaking in MCC. In both interneuron populations, these area-specific differences were statistically significant (for SOM: χ^2^ = 21.4, p < .001, for PV: χ^2^ = 25.13, p < .001). The same overall pattern was visible when jointly considering all BALB/cJ mice (cross-area differences for SOM: χ^2^ = 15.0, p = .002; for PV: χ^2^ = 20.64, p < .001), although both the overall number of interneurons and their area-specific distribution seemed to be subdued. The only significant difference between the interneuron distributions of BALB/cJ and BALB/cByJ mice was the reduced number of PV interneurons in A32 of ACC (U = 22.0, p = .043) and in MCC (U = 7.0, p = .003) of BALB/cJ mice.

However, distribution differences became more pronounced when examining highly-sociable and less-sociable BALB/cJ mice separately (**Figure 3B**). Strikingly, for both SOM and PV interneurons, less-sociable BALB/cJ mice showed an essentially flat distribution of interneurons across cingulate cortex (all χ^2^ < 6.6, all p > .08). While in PV interneurons, this also translated to a decreased overall density across all four cingulate areas compared to control BALB/cByJ mice (all U < 5, all p < .05), in SOM interneurons, the overall numbers did not differ significantly, only their distribution across areas. In contrast, highly-sociable BALB/cJ mice showed intermediate interneuron distributions that were overall more akin to those in BALB/cByJ mice: the area-specific distribution of SOM and PV interneurons was largely preserved (all χ^2^ > 8.8, all p < .05), and while PV interneuron counts were lowered across all cingulate areas, a significant decrease compared to BALB/cByJ mice only occurred in MCC (U = 6, p = .02). This suggests that a differentiated distribution of SOM and PV interneurons across cingulate cortex may be required in order to express normal levels of social interest while decreases in MCC PV populations seem to be mostly related to aggressive behavior. For correlations of behavioural read-outs and interneurons across ACC/MCC see **Supplementary Material Tables 1** and **2**.

## Discussion

Here we have shown, for the first time, that aggression and sociability in BALB/cJ mice are linked to the differential distribution of PV and SOM interneurons across ACC and MCC, suggesting that a specific balance of inhibitory control across cingulate cortex may be required to successfully regulate these behaviors. Compared to BALB/cByJ mice, BALB/cJ mice show elevated levels of aggression, both in socially acceptable and anti-social forms. In terms of sociability, we encountered an interesting behavioral divergence in BALB/cJ mice: one group of BALB/cJ mice showed strongly reduced levels of social interest in the 3CT, as well as lower levels of socially acceptable aggression. In contrast, the second group of BALB/cJ mice demonstrated as much, if not more, social interest in the 3CT as control animals, but also as much, if not more, overall aggression as less-sociable BALB/cJs. These behavioral phenotypes were in turn associated with specific distributions of PV and SOM interneurons across ACC and MCC: for control animals, PV interneuron density increased largely linearly across cingulate cortex, while SOM interneurons were mostly concentrated in A25 of the ACC and in MCC, but rarely found in A32 of the ACC. In contrast, less-sociable BALB/cJ mice showed a nearly flat distribution of PV and SOM interneurons across cingulate cortex, with uniformly reduced levels of PV interneurons. Interestingly, highly-sociable BALB/cJ mice had an interneuron distribution that was intermediate but overall more similar to control mice than less-sociable BALB/cJ mice (see **Figure 3B**). Specifically, both BALB/cByJ and highly-sociable BALB/cJ mice showed significant area-specific differences in the concentration of SOM and PV interneurons, but less-sociable BALB/cJs did not. While for PV interneurons, the flattened distribution observed in less-sociable BALB/cJ mice is also accompanied by a decrease in the overall number of neurons, for SOM interneurons, animals did not differ in the overall amount of neurons but only in the specificity of the distribution. This suggests that sociability may rely on a differentiated distribution of (SOM and PV) interneurons across ACC and MCC.

In contrast, the regulation of aggression seemed to mainly relate to the number of PV interneurons, particularly in MCC: the only anatomical difference between highly-sociable BALB/cJ and control BALB/cByJ mice was a reduction in PV interneurons in MCC. This suggests that a sufficiently high concentration of PV interneurons in MCC may be particularly crucial to successfully regulate aggression. Together, these results add further evidence that cingulate cortex is indeed involved in the regulation of both aggression and sociability, possibly with a stronger involvement in sociability. Note that none of the behavior-related anatomical differences highlighted here would have been observed if rather than using the definition of ACC and MCC which is homologous to that in higher mammals (57), one were to partition cingulate cortex into cingulate area 1/cingulate area 2 (Cg1/Cg2), as has been classically applied in mice (see 31 for further analysis on this point). These results also extend the concept that a loss of GABAergic control mechanisms within cingulate cortex impedes the regulation of social behavior and aggression in BALB/cJ mice (36). Our results indicate that not only the overall availability of GABAergic inhibition seems to be crucial, but also its specific distribution across interneuron types and sub-areas of cingulate cortex. We previously reported volumetric alterations in ACC/MCC of BALB/cJ mice, demonstrating an increase in ACC volume but decrease in MCC volume in aggressive BALB/cJ mice compared to BALB/cByJ mice (31). In the current study, we observed that PV interneurons in BALB/cJ mice are decreased particularly in MCC, suggesting that the volume reduction of MCC amplifies the observed difference in terms of absolute numbers of PV interneurons. While we observed an increase in ACC volume previously and now decreases in interneuron populations, this suggests that the total number of interneurons within ACC might be similar for both mouse strains, but in BALB/cJ mice, interneurons would still be scattered less densely through a larger volume of brain tissue.

The current study highlights correlative links between the interneuron distribution of cingulate cortex and aggressive and sociable behavior, without testing their causal relations directly. While it is therefore difficult to estimate the strength of potential causal links, we can at least make confident assumptions about the direction of causality. Highly and less-sociable animals, as well as more and less aggressive animals, will most likely encounter somewhat different experiences (due to their own behavior and other animals’ reactions to their behavior), both throughout their life time and during the RI test and 3CT. Such experiences are likely to modulate both the connectivity and activity patterns of neuronal populations, including within cingulate cortex (58–60). However, it is unlikely that such experiences (and especially temporary experiences as the RI test or the 3CT) would have resulted in the systematic cell death of PV and/or SOM populations. For example, even in a much more drastic case—when visual input to visual cortex is entirely discontinued—neurons in visual cortex do not die but are instead repurposed (61). Therefore, it is much more likely that cell death of PV and SOM populations causes the observed changes in behavior than vice versa. Of course, demonstrating this directly would require developmental studies of PV and SOM populations in cingulate cortex.

In terms of the strength of a potential causal relation, it is important to note that the failed regulation of aggressive as well as asocial behavior likely hinge on a wide range of structural and functional changes, including the interneuron balance in other regions—for instance in the amygdala, given its role in the threat circuitry (e.g., 62, 63). While further characterization of such changes across different brain regions is needed, the results presented here highlight the fact that the differential distribution of interneurons across sub-regions of cingulate cortex is a much better predictor of behavior than the overall concentration of inhibitory neurons throughout cingulate cortex. For example, the use of the Cg1/Cg2 definition would have made it impossible to pinpoint PV interneuron concentration in MCC as the strongest predictor of aggression—after all, MCC does not exist in the Cg1/Cg2 definition. Likewise, differences in PV and SOM populations across A25 and A32 of ACC would not have been studied with the Cg1/Cg2 definition given that this definition does not include these two regions as part of the ACC. These results emphasize the need to treat cingulate cortex not as one homogeneous structure, but rather study the different sub-regions of cingulate cortex and their contribution to behavior separately.

What could be the functional significance of the interneuron distributions we observe? This question will only be answered conclusively by *in vivo* studies, particularly in behaving animals. However, based on existing literature we can make some educated guesses. Maintaining cortical circuit functioning depends to a large extent on an optimal balance between excitation and inhibition (E/I balance); and PV and SOM interneurons play an important role in maintaining this balance (64–68).

Regarding the functional role of SOM interneurons within the microcircuit, they are thought to provide more prolonged and widespread inhibition to local principal neurons by gating the inputs arriving onto their distal dendrites, often based on input from long-range feedback connections (69–71). As such, SOM interneurons play an important role in filtering the inputs of long-range connections, which has in turn been directly related to the ongoing modulation of learning (72–74). The concentration of SOM interneurons in A25 and MCC as opposed to A32 could therefore result in stronger inhibitory control of autonomic circuitry [thought to be mediated by A25 (75)] and approach/avoidance selection [processed by MCC (76)] as opposed to fear expression [supported by A32 (76)]. In contrast, PV interneurons have been shown to provide fast inhibition to pyramidal cells (71). This not only suppresses but also temporally structures local network responses by driving oscillations, particularly in the gamma frequency range (77, 78). Such oscillations are in turn thought to play a prominent role in gating and integrating sensory information (79, 80), thereby enhancing cortical circuit information processing (81). The increased presence of PV interneurons in MCC, which serves as a hub connecting ACC and retrosplenial cortex, could therefore help to coordinate information flow within cingulate cortex, streamlining the decision whether to approach or to avoid a social situation (24). This tallies with the finding that in human patients, decreases in the number of PV interneurons within prefrontal cortical regions have been documented in a number of clinical conditions involving deficits in social behavior, in particular autism (39), and schizophrenia (37). Consistent with this, recent reports have suggested that modulation of prefrontal cortex excitation/inhibition balance can rescue deficiencies in social behavior in CNTNAP2-deficient mice, underlining the crucial role of PV populations in these behaviors (82). Together, these studies suggest that a finely balanced distribution of PV and SOM across cingulate cortex is likely needed to coordinate the precisely timed cross-cingulate neuronal dynamics that generate social interaction and aggressive behavior. As such, our findings provide intriguing area-specific predictions for *in vivo* studies testing the network dynamics underlying the ongoing regulation of aggressive and social behavior.

In terms of cognitive processing, one possible interpretation of the fact that the interneuron distribution of highly-sociable BALB/cJs neither fully resembles that of BALB/cByJs nor that of less-sociable BALB/cJs, is the following: aggression and social avoidance are actually not independent behavioral processes, but form parts of the same spectrum spanning different levels of ability to navigate social situations. Analyzing and responding to social interactions arguably requires intricate and well-balanced neuronal interactions, for instance within cingulate cortex. When this balance is upset moderately, e.g., due to modest deviations from the ideal anatomical interneuron distribution, animals may exhibit increased aggression—which can be seen as a simplistic, coarse solution to social interactions (highly-sociable BALB/cJ mice). As the anatomical structure deviates further from its ideal balance, animals begin to avoid social contact altogether (less-sociable BALB/cJ mice), but in the event of unavoidable contact immediately resort to extreme aggressive responses—hence the identical amount of anti-social, but not socially acceptable, aggression between highly-sociable and less-sociable BALB/cJ mice.

The divergence between highly-sociable and less-sociable BALB/cJ mice might also map onto the sub-groups observed within patients with CD. Within CD patients, one group with so-called callous-unemotional traits shows vastly reduced amounts of empathy, favors rational over emotional information in social decision making (6), and in addition to showing “hot-blooded” impulsive aggression (i.e., spontaneously reacting to a perceived threat) engages also in instrumental “cold-blooded” aggression [i.e., planned, calculated acts of aggression (83)]. In contrast, the group without callous and unemotional (CU) traits shows more reactive “hot-blooded” aggression, and less signs of instrumental aggression. Translating this to the sub-groups we observed within our BALB/cJ mice, highly-sociable BALB/cJ mice may perceive the other animal as a threat (both during the 3CT and the RI test), resulting in impulsive bursts of aggression, both in the form of socially acceptable and less acceptable aggression. In contrast, less-sociable BALB/cJ mice do significantly engage less in acceptable aggressive behavior: they do not warn the intruder mouse, neither in form of threats nor back bites. Instead, they immediately attack vulnerable body parts, which might translate less to reactive and more to instrumental aggression. Subgrouping of BALB/cJ mice has also been suggested elsewhere (83) in the context of low and high-empathy like behaviors. This reinforces our concept of subgrouping BALB/cJ mice according to their aggressive and social phenotypes. It may further suggest that those less-sociable BALB/cJ investigated here may be comparable to the low-empathy like BALB/cJ phenotype documented elsewhere (84, 85) and therefore relevant to the study of CU traits.

One might argue that decreased sociability might be related to, or even a symptom of, other behavioral traits. One of the most likely candidates for such a scenario is increased anxiety, which could cause an aversion to social contact. Indeed, BALB/cJ mice are generally known to be more anxious than BALB/cByJ mice (34, see also **Supplementary Material Figure 2**). However, we can exclude differences in anxiety between highly-sociable and less-sociable BALB/cJ mice as a possible confound: all BALB/cJ mice tested here were more anxious than BALB/cByJ mice, but less-sociable BALB/cJ mice were not more anxious than highly-sociable BALB/cJ mice (**Supplementary Material Figure 2**). In addition, differences in sociability are unlikely to be related to general learning ability: recently it was demonstrated that BALB/cJ mice show a largely uniform level of performance in terms of learning a simple appetitive task, which was also comparable to that of BALB/cByJ mice (86); if at all, BALB/cJ mice seemed to be somewhat more reward-driven than BALB/cByJ mice, however there were no apparent sub-groups or large in-group variations regarding this trait. Of course, there are many more behavioral traits that could show a relation to the observed sub-grouping based on sociability within the BALB/cJ strain. Future work is needed to disentangle how differences in aggression and sociability map onto other behavioral traits, both in terms of behavioral sub-groups and neuronal correlates.

In conclusion, our study demonstrates for the first time that a precise balance of specific interneuron types across sub-areas of cingulate cortex may be required to regulate complex behaviors such as aggression and social contact. This insight expands on—and provides a link between—findings showing a role of ACC and MCC in both aggression and social behavior in human patients (3, 56, 87–89) as well as rodent data showing that cortex-wide changes in PV and SOM interneuron populations are related to psychiatric symptoms (40–43). Our results also reinforce the notion that different areas of cingulate cortex are best considered as functionally separate entities that drive specific neurobehavioral dynamics. Finally, our findings provide important constraints and predictions for future *in vivo* studies into the functional role of SOM and PV interneuron activity within cingulate cortex.

## Data Availability Statement

The datasets generated for this study are available on request to the corresponding author.

## Ethics Statement

All applicable international, national, and/or institutional guidelines for the care and use of animals were followed. The current research was performed under the approval of the Ethics Committee on Animal Experimentation of Radboud University (RU- DEC number 2014-149) and the European Directive 2010/63/EU.

## Author Contributions

SH designed and performed the experiments, analyzed the data and wrote the manuscript. FM performed experiments. MW performed experiments and analyzed data. FG analyzed data. AF provided feedback on the manuscript and graphs. JB and CB provided feedback on the manuscript. JG wrote the manuscript. MH designed the experiment, analyzed data and wrote the manuscript.

## Funding

SH has been supported by a Donders Institute Top Talent grant. The research leading to these results has received funding from the European Community’s Seventh Framework Programme (FP7/2007-2013) under grant agreement no. 602805 (Aggressotype) and no. 603016 (MATRICS) to JB and JG. Further, this research has received funding from the Innovative Medicines Initiative 2 Joint Undertaking under grant agreement no. 115916 (PRISM) to CB and JG. This Joint Undertaking receives support from the European Union’s Horizon 2020 research and innovation programme and EFPIA.

## Conflict of Interest

JB was a consultant to/member of advisory board of/and/or speaker for Janssen-Cilag BV, Eli Lilly, Takeda (Shire), Medice, Roche and Servier. JG has in the past four years been a consultant to Boehringer Ingelheim GmbH. Neither JB nor JG are employees of any of these companies, and neither are stock shareholders of any of these companies. The funding organizations or industrial consultancies listed have had no involvement with the conception, design, data analysis and interpretation, review and/or any other aspects relating to this paper.

The remaining authors declare that the research was conducted in the absence of any commercial or financial relationships that could be construed as a potential conflict of interest.

The handling editor declared a shared affiliation, though no other collaboration with the authors.
