# Supplementary material for: Gradient of Parvalbumin- and Somatostatin-Expressing Interneurons Across Cingulate Cortex Is Differentially Linked to Aggression and Sociability in BALB/cJ Mice
Source: Front Psychiatry. 2019 Nov 15;10:809. doi: 10.3389/fpsyt.2019.00809 (PMC6873752; doi:10.3389/fpsyt.2019.00809)
Supplement: Supplementary file 1 [file DataSheet_1.docx]

*Supplementary Material Table 1:* Behavior & Interneuron correlation for BALB/cJ mice

|  | **Sniffing time social cylinder** | **Sniffing time anti-social cylinder** | **SOM A25** | **SOM A32** | **SOM A24** | **SOM MCC** | **PV**  **A25** | **PV**  **A32** | **PV**  **A24** | **PV**  **MCC** |
| --- | --- | --- | --- | --- | --- | --- | --- | --- | --- | --- |
| **Bites 3 days** r  p | .652  .384 | -.187  .800 | -.615  .400 | -.459  .430 | -.173  .806 | .626  .384 | .100  .922 | .317  .631 | .557  .400 | .287  .658 |
| **Bites 5 days** r  p | .563  .400 | -.082  .943 | -.619  .398 | -.478  .430 | -.280  .664 | .650  .384 | .238  .723 | .207  .763 | .643  .384 | .211  .763 |
| **Attack** r  **latency 3 days**  p | -.547  .400 | .382  .538 | .570  .404 | .709  .384 | -.073  .952 | -.291  .658 | -.029  .993 | -.433  .457 | -.476  .430 | -.462  .430 |
| **Attack** r  **latency 5 days**  p | -.471  .430 | .358  .576 | .647  .384 | .659  .384 | .014  .993 | -.415  .466 | -.046  .975 | -.428  .457 | -.605  .384 | -.293  .658 |
| **Anti-social** r  **attacks 3 days**  p | .494  .430 | -.220  .752 | -.587  .398 | -.295  .658 | -.237  .723 | .691  .384 | -.048  .975 | .241  .723 | .346  .593 | .060  .960 |
| **Anti-social** r **attacks 5 days**  p | .445  .441 | -.009  .992 | -.689  .384 | -.422  .462 | -.363  .576 | .605  .384 | .157  .810 | .126  .875 | .548  .398 | -.018  .993 |
| **Sniffing time** r **social cylinder**  p | N/A | .164  .806 | -.190  .806 | -.269  .680 | -.454  .431 | .497  .430 | .464  .430 | .541  .430 | .507  .430 | .492  .430 |
| **Sniffing time** r **anti-social cylinder** p | .164  .806 | N/A | -.776  .384 | -.002  .997 | -.743  .384 | -.326  .620 | .471  .430 | -.096  .922 | .021  .992 | -.337  .606 |

*Note*. Correlations are Pearson correlation coefficients; correlation is significant at the .05 level (two-tailed). All correlations corrected with the false discovery rate.

*Supplementary Material Table 2:* Behavior & Interneuron correlation for BALB/cByJ mice

|  | **Sniffing time social cylinder** | **Sniffing time anti-social cylinder** | **SOM A25** | **SOM A32** | **SOM A24** | **SOM MCC** | **PV**  **A25** | **PV**  **A32** | **PV**  **A24** | **PV**  **MCC** |
| --- | --- | --- | --- | --- | --- | --- | --- | --- | --- | --- |
| **Bites 3 days** r  p | .231  .964 | .520  .964 | -.326  .964 | -.069  .991 | -.099  .977 | .018  .999 | -.243  .964 | -.284  .964 | .015  .999 | -.185  .964 |
| **Bites 5 days** r  p | .475  .964 | .138  .964 | -.209  .964 | .010  .999 | .261  .964 | .294  .964 | -.235  .964 | -.600  .964 | -.139  .964 | -.069  .991 |
| **Attack** r  **latency 3 days**  p | -.142  .964 | -.449  .964 | .280  .964 | .132  .964 | .194  .964 | .096  .977 | .194  . 64 | .304  .964 | .047  .999 | .267  .964 |
| **Attack** r  **latency 5 days**  p | -.357  .964 | -.249  .964 | .209  .964 | -.004  .999 | -.030  .999 | -.084  .980 | .271  .964 | .447  .964 | .091  .977 | .250  .964 |
| **Anti-social** r  **attacks 3 days**  p | .171  .964 | .524  .964 | -.329  .964 | -.103  .977 | -.132  .964 | -.023  .999 | -.216  .964 | -.309  .964 | .000  .999 | -.192  .964 |
| **Anti-social** r **attacks 5 days**  p | .554  .964 | -.007  .999 | -.172  .964 | .134  .964 | .387  .964 | .412  .964 | -.334  .964 | -.641  .964 | -.168  .964 | -.067  .991 |
| **Sniffing time** r **social cylinder**  p | N/A | -.398  .964 | .573  .964 | .680  .964 | .748  .964 | .452  .964 | -.283  .964 | -.253  .964 | .018  .999 | -.133  .977 |
| **Sniffing time** r **anti-social cylinder** p | -.398  .964 | N/A | -.451  .964 | -.277  .964 | -.291  .964 | -.104  .977 | .289  .964 | .262  .964 | .405  .964 | .205  .964 |

*Note*. Correlations are Pearson correlation coefficients; correlation is significant at the .05 level (two-tailed). All correlations corrected with the false discovery rate.
