# Supplementary figures and images for: Gradient of Parvalbumin- and Somatostatin-Expressing Interneurons Across Cingulate Cortex Is Differentially Linked to Aggression and Sociability in BALB/cJ Mice
Source: Front Psychiatry. 2019 Nov 15;10:809. doi: 10.3389/fpsyt.2019.00809 (PMC6873752; doi:10.3389/fpsyt.2019.00809)

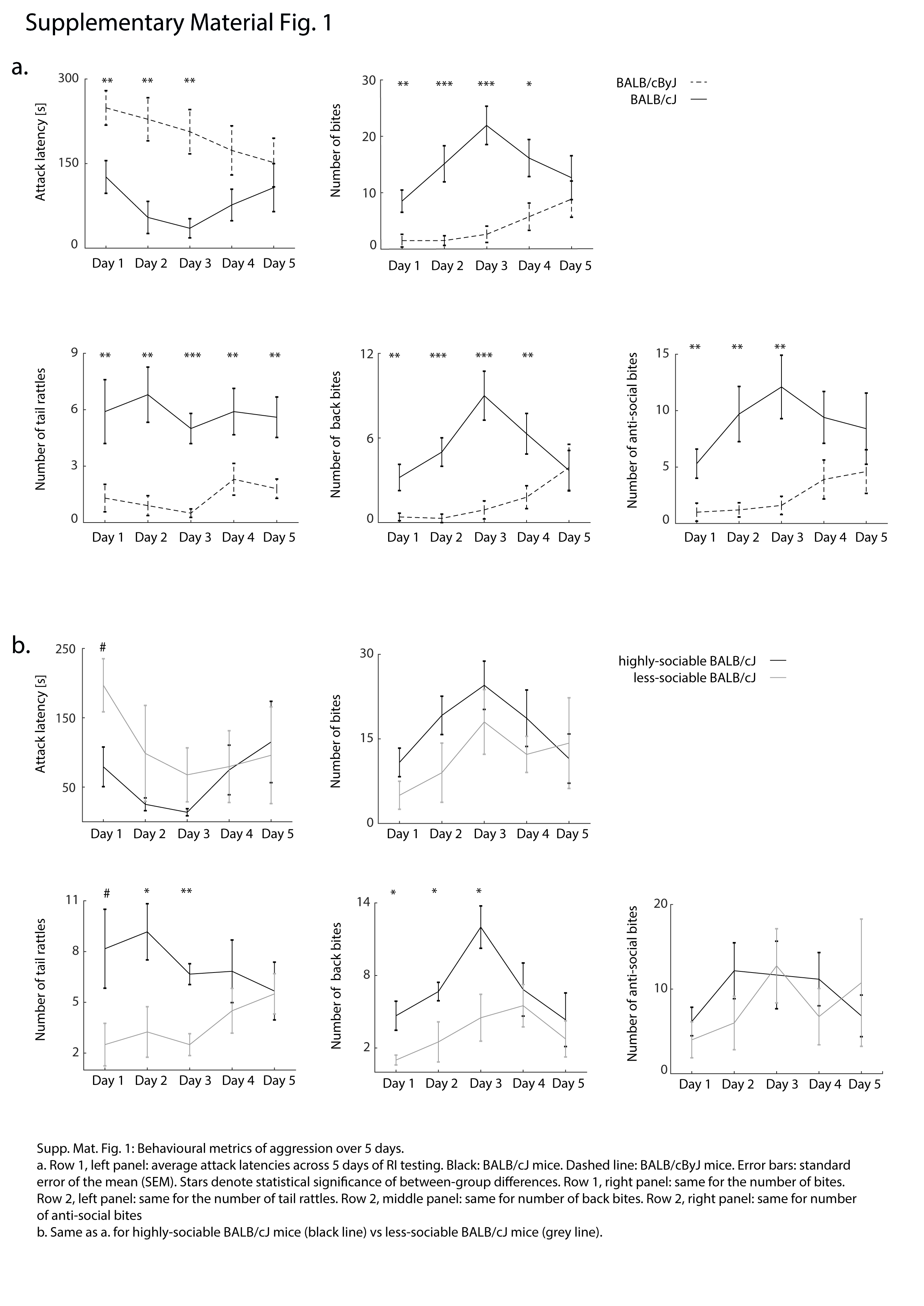

Supplement: Supplementary file 2 [file Image_1.tif]

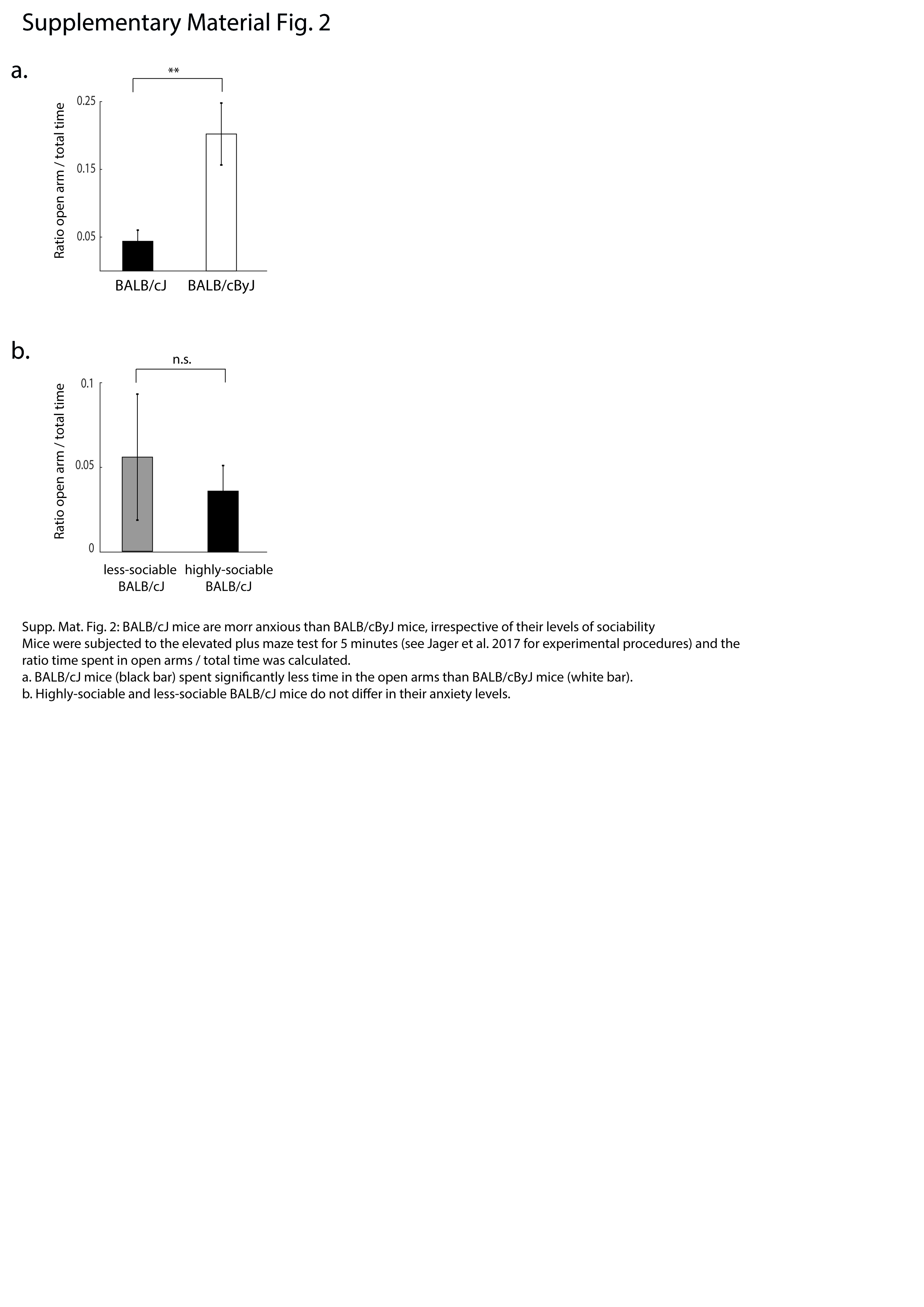

Supplement: Supplementary file 3 [file Image_2.tif]
